# Suspect Sweetener: Arsenic Detected in Organic Brown Rice Syrup

**DOI:** 10.1289/ehp.120-a204a

**Published:** 2012-05-01

**Authors:** Wendee Holtcamp

**Affiliations:** Wendee Holtcamp, based in Houston, Texas, has written since 1997 for *Nature*, *Scientific American*, and other magazines.

Organic brown rice syrup (OBRS) is a sweetener frequently used as an alternative to high-fructose corn syrup in organic and health food products. In a study of children’s foods, a team of researchers discovered high levels of arsenic in toddler formula products that contained OBRS. Given evidence that arsenic accumulates in rice at high levels, the researchers suspected OBRS was the source of the arsenic in the formulas—a suspicion corroborated by additional tests of several products both with and without the sweetener [*EHP* 120(5):623–626; Jackson et al].

The researchers tested 3 commercially available brown rice syrups, 29 cereal bars (18 with OBRS), 3 high-performance “energy shot blocks” with OBRS, 15 infant formulas without the sweetener, and 2 toddler formulas with it. The researchers measured inorganic arsenic as well as the organic chemical species dimethylarsenate (DMA) and monomethylarsenate (MMA), which are generally believed to be less toxic than inorganic arsenic.

**Figure:**
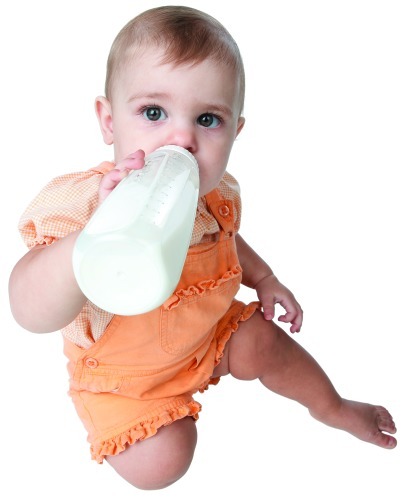
The two toddler formulas tested are the only ones known to be made with OBRS. © Getty Images

The OBRS-sweetened toddler formulas (one soy-based, one milk-based) had about 20 times the total arsenic concentrations of the non-OBRS infant formulas. Samples of prepared milk-based toddler formula had inorganic arsenic concentrations just below the current Environmental Protection Agency (EPA) drinking water standard of 10 µg/L, whereas inorganic arsenic in samples of soy-based toddler formula tested 1.5–2.5 times above the EPA standard.

In the brown rice syrups tested, inorganic arsenic made up 80–90% of the arsenic content of two of the syrups and half the arsenic content of the third. All three also contained DMA, with lesser amounts of MMA. All 29 cereal bars tested contained some arsenic, but those without any rice-based ingredients had the lowest levels. Nearly 60% of the others contained inorganic arsenic. The three energy shot blocks contained enough inorganic arsenic that if an individual were to consume the manufacturer-recommended four servings during a two-hour workout, they would ingest the equivalent of drinking a liter of water containing 10 µg/L arsenic; total arsenic would be twice that.

The two toddler formulas tested were the only ones the researchers could find that contained OBRS. However, they estimate that approximately half of all cereal and energy bars contain OBRS or other rice products, and they conclude that food containing OBRS may be a major pathway to arsenic exposure for some people. Although the health effects of low-level arsenic exposure over limited durations are unknown, childhood exposure may affect lifelong health and should be limited. The authors therefore urge U.S. regulators to establish limits for arsenic levels in food, particularly infant and toddler formulas.

